# Harm reduction and law enforcement in Vietnam: influences on street policing

**DOI:** 10.1186/1477-7517-9-27

**Published:** 2012-07-09

**Authors:** Melissa Jardine, Nick Crofts, Geoff Monaghan, Martha Morrow

**Affiliations:** 1 Nossal Institute for Global Health, The University of Melbourne, 4th Floor, 161 Barry Street, Carlton, VIC 3010, Australia; 2 Melbourne School of Population Health, The University of Melbourne, Melbourne, Australia; 3 Centre for Law Enforcement and Public Health, Melbourne, Australia

## Abstract

**Background and rationale:**

The HIV epidemic in Vietnam has from its start been concentrated among injecting drug users. Vietnam instituted the 2006 HIV/AIDS Law which includes comprehensive harm reduction measures, but these are unevenly accepted and inadequately implemented. Ward police are a major determinant of risk for IDUs, required to participate in drug control practices (especially meeting quotas for detention centres) which impede support for harm reduction. We studied influences on ward level police regarding harm reduction in Hanoi to learn how to better target education and structural change.

**Methods:**

After document review, we interviewed informants from government, NGOs, INGOs, multilateral agencies, and police, using semi-structured guides. Topics covered included perceptions of harm reduction and the police role in drug law enforcement, and harm reduction training and advocacy among police.

**Results:**

Police perceive conflicting responsibilities, but overwhelmingly see their responsibility as enforcing drug laws, identifying and knowing drug users, and selecting those for compulsory detention. Harm reduction training was very patchy, ward police not being seen as important to it; and understanding of harm reduction was limited, tending to reflect drug control priorities. Justification for methadone was as much crime prevention as HIV prevention.

Competing pressures on ward police create much anxiety, with performance measures based around drug control; recourse to detention resolves competing pressures more safely. There is much recognition of the importance of discretion, and much use of it to maintain good social order. Policy dissemination approaches within the law enforcement sector were inconsistent, with little communication about harm reduction programs or approaches, and an unfounded assumption that training at senior levels would naturally reach to the street.

**Discussion:**

Ward police have not been systematically included in harm reduction advocacy or training strategies to support or operationalise legalised harm reduction interventions. The practices of street police challenge harm reduction policies, entirely understandably given the competing pressures on them. For harm reduction to be effective in Vietnam, it is essential that the ambiguities and contradictions between laws to control HIV and to control drugs be resolved for the street-level police.

## Introduction

Where there is injecting drug use (IDU), there is risk of transmission of blood-borne viruses (BBV), including HIV and hepatitis B and C (HBV/HCV). Comprehensive harm reduction approaches have been convincingly shown to diminish this risk and to prevent BBV transmission. After the first HIV case was reported in Vietnam in 1990, the number of reported HIV infections grew rapidly in all provinces [1]. Through this time the major group at risk of HIV infection has been injecting drug users (IDUs); this continues to be the case [2]. In 2009, the Vietnamese Government estimated there were 150,000 drug users nationwide, of which 83 % were IDUs [3]. In 2007, WHO reported the national adult HIV prevalence as 0.5 % [4].

Key harm reduction measures enjoy official recognition and support in Vietnam through the *Law on HIV/AIDS Prevention and Control*[5] (hereinafter referred to as the 2006 HIV/AIDS Law). Nevertheless, these measures are unevenly accepted at grassroots level and inadequately implemented to have maximum impact on reducing HIV transmission among and from IDUs [6]. The 2006 HIV/AIDS Law describes ‘harm reduction intervention measures’ as including education, mobilisation, the encouragement of the use of condoms and clean needles and syringes, substitution therapy for opiate addiction and ‘…other harm reduction intervention measures in order to facilitate safe behaviours to prevent HIV transmission’ (Article 2.15). The Law was passed by the National Assembly of Vietnam, the highest level of government. The following year, Decree 108 [7], which provides details for the implementation of the 2006 HIV/AIDS Law, was passed ‘at the proposal of the Minister for Health’; Article 22 states that the Minister for Health is responsible for implementing the Decree. Although forming part of the legal framework for the health sector regarding HIV/AIDS, Decree 108 stipulates responsibilities for other sectors including the Ministry of Public Security (MoPS) and the government and Communist Party lowest bureaucratic level – the ward (urban) or commune (rural) level. Specifically, Decree 108 states that the ward or commune level People’s Committee (community level arm of the Communist Party) and police must be notified prior to the implementation of any harm reduction intervention in their jurisdiction, and these actors share responsibility for ‘…creating favourable conditions for programs and projects to operate…’ The implication is that ward police (also referred to in this paper as ‘street police’ due to the nature of their work taking place at the street or community level) and the People’s Committees must not only be notified of planned interventions, but also have access to harm reduction information in order to fulfil these responsibilities.

MoPS is responsible for the police, drug control and administrative bodies; injection of illicit drugs means that MoPS is a major influence on injecting behaviour and HIV risk among IDUs. Approaches to minimise HIV risk among IDUs must therefore involve MoPS. The National Strategy for HIV/AIDS Prevention and Control [8], which preceded the 2006 HIV/AIDS Law, described the model of ‘Ward policemen to participate in HIV/AIDS prevention and control’ as a typical activity, formulated and implemented by the MoPS. However, to date there is a lack of documentation about what this model looks like or how it is carried out at the operational policing level. The 2006 HIV/AIDS Law highlighted the need to link HIV/AIDS prevention with high-risk behaviours such as drug use and sex work through their ‘prevention and control’, but added that importance should be attached to harm reduction intervention measures (Article 3.3).

From a law enforcement perspective, there are two prevailing legal documents which pertain to the implementation of harm reduction approaches, namely, the Law on Drug Prevention and Control 2000 [9] (amended in 2008 to include explicit support for harm reduction interventions) [10] and the Ordinance for Handling Administrative Violations 2008 (OHAV) [11]. Draconian drug control policies and practices present major impediments to effective harm reduction services, which need to operate in environments in which clients feel confident that accessing services will not expose them to police harassment, arrest and incarceration [12]. Draconian approaches also tend to push drug use further underground, where needle sharing becomes more common, with related increased BBV transmission risk. The proximate agent of this influence is the law enforcement sector, in particular, for the IDU on the street, the police. Some studies have indicated that police behaviour may be one of the strongest determinants of IDU HIV risk behaviour [13], in addition to illicit drug laws and policies which contribute to the macroenvironmental risk factors [12].

In Vietnam, many IDUs are sent to compulsory detention centres (also referred to as ‘06 Centres’) to undergo forced detoxification. The number of people sent to these centres is largely dependent on a system which requires police to meet a set quota, although some, albeit very few, go voluntarily. Forced detoxification in compulsory detention fails to conform to evidence-based drug treatment approaches [6] although the police are still required to send IDUs to them based on annual Government directed Action Plans.

Drug control laws are frequently cited as barriers to effective harm reduction interventions [14], although the published literature offers little by way of explication as to why police services and other law enforcement agencies choose to support or impede the lawful implementation of harm reduction policies and practices. Previous studies in Vietnam have been critical of police behaviour towards IDUs, which is seen to impair confidence in accessing harm reduction services [15]. Some studies, however, reported positive relationships between IDUs and police [16][17]; these and other studies have recognised that engaging with the law enforcement sector is essential to successful outcomes for harm reduction approaches [18]. Like a number of countries, Vietnam is grappling with diverse and conflicting pressures in developing its HIV prevention approaches. The law enforcement sector is clearly critical to effective HIV prevention, but there is a gap in understanding the influences on and processes of change within the sector.

Importantly, harm reduction advocacy or training for police must be targeted at those who are empowered to assist in harm reduction implementation, whether through policy design or grassroots practice. Targeting harm reduction advocacy at senior police with a view to attaining whole of law enforcement sector support is, in theory, an appropriate objective; however, it is yet to be clearly demonstrated how police or policy makers in Vietnam, who are aware of harm reduction, translate knowledge or expectations of behaviour to subordinate levels of police for implementation.

In many policing jurisdictions, it is common for police recruits to be trained at and graduate from a single type of training institution. In Vietnam, there are three police training institutions; acceptance at a particular institution determines the level at which police will work, with each level having a corresponding seniority and status in the hierarchy. Lower results in competitive examinations gain entry into the Police College (a training institution for ward police), which offers just three years’ training, versus the Police Academy, which is five years and bestows the equivalent of a university degree. Police College graduates end up serving at the lowest administrative level, the commune (rural) or ward (urban) – the equivalent of ‘street police’, and the bottom of the hierarchy. Academy graduates are destined for district level or above, which offers far greater scope for promotion and specialisation. The third training institution for police has greater focus on policy than operational police duties and is perceived to be superior to both the College and Academy.

The study reported here explores to what extent and in what ways harm reduction advocacy has influenced police practice at street level (commune or ward) in Vietnam. This research seeks to bring a deeper understanding of the structural and cultural conditions facing police and the impact of these on decision-making processes.

## Methods

A range of methods was used, including document review, key informant interviews and a survey. Document review was used to identify the legal and policy framework relevant for our investigation. Principal documents included:

· 2006 HIV/AIDS Law

· Law on Drug Control 2000 (amended 2008)

· National Strategy on HIV/AIDS 2004

· Decree Detailing the Implementation of a Number of Articles of the Law on HIV/AIDS Prevention and Control (Decree 108) 2007

· The Law on People’s Public Security Forces 2005

· Ordinance on Commune Police 2008; and,

· Ordinance on Handling Administrative Violations (OHAV) 2008.

A total of 36 individual interviews using semi-structured guides was conducted to investigate factors that facilitate or impede police support for harm reduction. Informants were identified from a range of key government policy areas, in addition to relevant international agencies and local non-governmental organisations (NGOs). Thirteen interviews were with police, including those from ward or district level within two districts of Hanoi, central level (serving and retired) and international representatives from law enforcement agencies*.* Topics covered in key informant interviews included: perceptions of harm reduction approaches and the role of police; the internal organisational structure of police in Vietnam; general police training and recruitment practices; and the nature of harm reduction training and advocacy to garner support among police.

Given that the conflicting HIV/AIDS Law and Drug Control Laws may have elicited criticism of government policies, interviews were not tape recorded. An interpreter was used and hand written notes were made for interviews with Vietnamese police.

A structured, self-administered questionnaire was used to survey 27 police working at street level in two Hanoi districts with concentrations of drug use (21 police from the Division of Ward Police [District level], District A, and 6 ward level police, District B).

Transcripts of interviews were prepared and analysed using thematic analysis, while simple descriptive statistics were derived from the survey using Microsoft Excel. All data sets were further analysed using Chan’s interactive model of the production of police practice [19]. This model posits that police should be regarded as ‘actors’ who experience a range of influences at a micro and macro level producing complex, dynamic, and, at times, unpredictable outcomes with respect to police reform and behaviour change.

## Results

Results are presented according to the study objectives, drawing on different data sets as appropriate.

### Police role in and understanding of harm reduction at the ward level

In order to examine the police role in harm reduction at the ward level we must first describe the role of ward police in general. These officers fulfil duties performed by community-beat police, street police, front-line police or general duties police in other jurisdictions, as well as functions that are specific to the drug law enforcement context in Vietnam. The Ordinance on Commune Police 2008 [20] outlines the tasks of ward police, which include, inter alia:

· *. . . apply measures to prevent and combat crimes and other law violations related to security, social order and safety …* (Article 3.2)

· *… to manage persons under special amnesty, drug-detoxified persons and persons having completely served their prison terms and being subject to further management according to law…* (Article 9.3)

· *…to enforce the law on residence management, people's identity cards and other travel papers…* (Article 9.5)

· *…to body-search, check belongings and personal papers and seize weapons or murder weapons of persons who are caught red-handed in committing illegal acts… (Article 9.6)*

· *…organize the [protection] of victims… (Article 9.6)*

· *…protect the scenes [of crimes]… (Article 9.6)*

· *…make initial records, take testimonies of victims and witnesses (Article 9.6)*

· *…to seize and preserve material evidences… (Article 9.6)*; and,

· *To sanction administrative violations; make dossiers proposing the application of other administrative sanctions against violators…* (Article 9.8).

According to one Ward Police Chief, “Drugs are only a portion of our work. We deal with terrorism, traffic, fraud, robbery, counterfeit money and fake products.” Another Ward Police Officer described his roles this way:

*The main one is to learn by heart the [identity and circumstances of the] 2000 people [in the ward]; second, to go to their houses and learn about updates, what is happening. Also we must compile profiles on the businesses, companies and shops … Also, do some analysis of [our local statistics] and give it to the district level. Sometimes we are messengers, giving out district level information to the local people, sometimes trying to do fundraising for poor people to raise money, such as for people affected by the floods. Sometimes there will be a campaign to vaccinate the dogs and cats for rabies or diseases. On the night shift sometimes we have to go out to deal with fighting between husbands and wives.* (Ward Police Officer 1, District B)

With the introduction of harm reduction interventions in Vietnam, the role of ward police in maintaining drug user profiles – which form the basis of decisions about compulsory residence in 06 centres – conflicts with their harm reduction implementation responsibilities as stipulated in Decree 108 [7], which requires them to support an enabling environment for the 2006 HIV/AIDS Law.

Whilst there has been no systematic approach to harm reduction training for ward police, they have been trained in some locations through programs supported by international agencies. Several police interviewees at the ward and district level said that the ward police were important to harm reduction but they were rarely, if ever, the target of training or advocacy to garner their support for harm reduction approaches.

*There are no classes or no training about this at the Police College. I just heard about the programs. I haven’t been trained. I learned from mass media, and other ward police haven’t been given any training.* (Ward Police Officer 1, District B)

Some police interviewees indicated that while familiar with the term ‘harm reduction’, they did not know what it meant in practice for police. Knowing that IDU is a major driver of the HIV epidemic in Vietnam, some police described their role in HIV prevention as being the prevention of drug use and monitoring of drug users. Although the 2006 HIV/AIDS Law states that infected people have the right to privacy and their sero-status being kept confidential (Article 4.d), one District Police Chief, who claimed to have been trained in harm reduction policies, stated that ward police bear the greatest responsibility for HIV prevention, part of which was “…to review the names of drug users and which ones have HIV”. He added that the focus of the HIV/AIDS Steering Committee of Hanoi was to reduce drug use in order to reduce HIV. One ward police officer (2, District B) said the role of police in HIV prevention was ‘clear’ but followed that with comments suggesting this meant drug law enforcement. Most police interviewed said that monitoring and conducting surveillance on drug users was a pervasive police activity.

*The police need to know who is in the [local drug users’ support program directed by the local authorities], their phone number, where they live and so on, so that they know who they need to keep under surveillance. Also, the police help the drug users [after they are released from the 06 centre], give them [encouragement] and consult with their family to help them prevent drug use again and to help them get a job.* (Ward Police Officer 1, District B)

Police around the world use a variety of methods to gather intelligence about criminal activities. The following quote describes a surveillance technique used by Vietnamese police for drug law enforcement.

*The [ward] police mostly sit on the coffee corner and pretend they are reading a book or the paper, but they are really listening to people talk and finding out about things. Many criminals hang out on the coffee corner. The police wear plain clothes for this.* (Central level Police Officer B)

**Figure 1:**
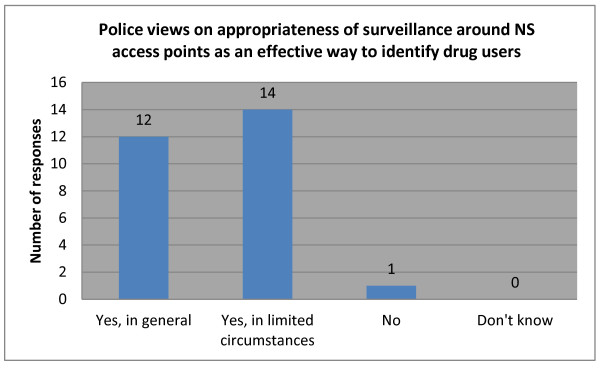
Police views on appropriateness of surveillance around NS access points as an effective way to identify drug users.

Although aware of the legality of harm reduction approaches such as needle and syringe programs (NSPs), some police explicitly stated they did not support NSPs, and instead exploited the existence of sites where NS are obtained as opportunities for police to identify IDUs (see Figure 1 below).

*Most of the activities of police [drug law enforcement is that] they will catch drug users who just wander around, but we have programs to use surveillance of pharmacies to watch the drug user buy needles and syringes. Some pharmacies sell needles and syringes to drug users and some don’t … The ward police have no responsibilities for needle and syringe programs. Even if a program was started here I would still not be in favour of this … I will surely never be in favour of such a program because it also makes drug users need drugs more.* (Ward Police Chief, District B)

**Figure 2:**
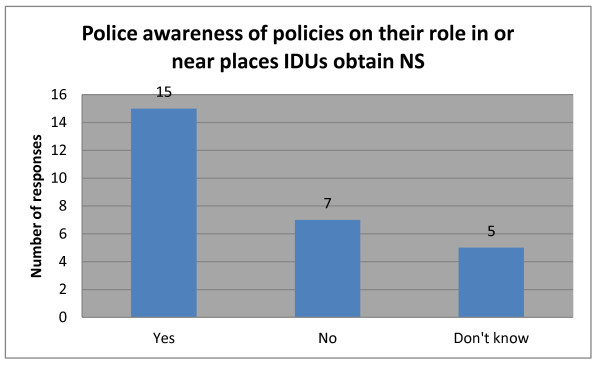
Police awareness of policies on their role in or near places IDUs obtain NS.

When asked if police were aware of any policies regarding their role in or near places to procure NS, over half of respondents reported that they were (Figure 2). The survey then provided a space for respondents to ‘please briefly summarise the policy.’ Only six wrote out the requested summary, which in every case related to the role of police in collecting used NS or organising the community to do so. Examples include: *“Conduct frequent surveillance all areas where drugs are used and then locate and seize syringes*”; and “*Getting all organisations and associations to collect all needles and syringes in the community according to [laws on] drugs and prostitution control*”.

**Figure 3:**
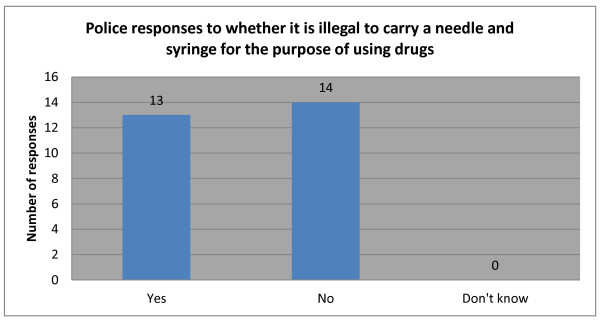
Police responses to whether it is illegal to carry a needle and syringe for the purpose of using drugs.

It is clear from the previous responses and Figure 3 that many police lack clear understanding of the Law on HIV/AIDS 2006. For example, almost half believed it was illegal to carry a needle and syringe for the purpose of injecting drugs although Articles 4.2 and 9 of Decree 108 (the implementing guidelines of the 2006 HIV/AIDS Law) expressly allow for this. To be fair, some of the respondents in answering this question might have had in mind Article 46 of the 2008 OHAV which makes it an administrative violation to possess materials or things used to facilitate an administrative violation which includes drug use.

**Figure 4:**
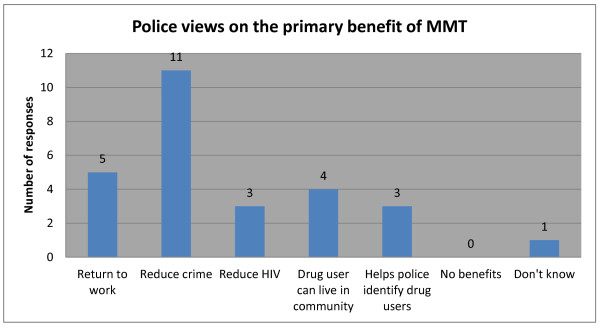
Police views on the primary benefit of MMT.

With respect to methadone maintenance therapy (MMT), although most police were aware of the therapy’s benefits in reducing HIV risk through reduced needle sharing among IDUs, almost half (11) of police surveyed saw the primary benefit of MMT as the subsequent reduction in crime, rather than a reduction in HIV risk (see Figure 4 below)

### Influences on police behaviour regarding drug law enforcement and harm reduction

Police behaviour with respect to drug law enforcement is regulated not only by legislation but a variety of factors which have a bearing on job security and promotion, including quotas for arrests, career reputation and internal police disciplinary procedures. The way these factors create anxiety is evident from the following quote, which relates to fulfilment of a key ward police duty: knowledge of residents in their jurisdiction.

Because there is a clear regulation for police that each ward police officer has to take care of 500 houses and about 1-2000 people, we must know and profile all the people. If we don’t know one house or one person we will be punished by the [district] police… Annually there is a team set up by the district to [audit our registrations]. If a ward police officer can’t meet his requirements he will be forced to quit his job. The number of warnings a police officer might get is not clear.

*A typical way an officer could lose his job is when a drug addict has been arrested by another police officer and [it became clear] the ward police didn’t know that person.* (Ward Police Officer 1, District B)

Pressure on police to identify and arrest drug users in their area was described as a stressor for ward police.

*Actually, the ward police don’t really like [crack-down] campaigns but we must do it. We are under pressure. It is hard work. Because nowadays the criminals know the campaigns, they know when they happen so they keep a low profile; so do the drug users. They can hide in another ward. In some campaigns, ward police in another ward or district might catch the drug users from your own ward who are hiding there. If they haven’t been profiled [by you] it’s a big problem. It is our biggest fear.* (Ward police officer 1, District B)

Quotas for arresting IDUs and sending them to detention centres are part of the performance measurement system. These apply both to individual police and to the ward as a whole.

*[If we are unable to meet the quota, the ward police] get no more awards, but the focus will be on the capacity of the Ward Chief… If the [quota] is not met for many years and [the ward police] can’t achieve their action plan the Chief will be rotated to another place and replaced by a new Chief. That’s why every year the Chief decides each person’s action plan. Each person will be allocated three cases. But the more [arrests] you make the more prestige for the officer, so everybody is trying to do their best.* (Ward police officer 1, District B)

Opportunities to be recognised for their performance through receiving certificates of merit for drug arrests may also prompt police to take action against drug use.

*Happiness at work is when police can feel good because they have [conducted a good investigation]; they can feel good about it for years. Vietnamese police sometimes get a certificate for good work which is a kind of benefit....*(Ward police officer 2, District B)

It is not just performance measurement indicators that may influence police behaviour regarding drugs, users and harm reduction. During key informant interviews, some police reported that discretion was used at times for the following reasons: police were too busy and had to prioritise responding to more serious offences, police regarded the drug user as being from a ‘good’ family or that the drug user was not ‘causing problems’ in the community. As the following quote illustrates, local police do have some discretion, which almost all police interviewed agreed was commonly used. Discretion is particularly relevant for the critical domain of police-community relationships; this emerges in decisions not to enforce the law in particular cases, especially first-time offences considered lesser infractions.

*One of the important things [to note is], we just use the law and punish people when the issue is serious. We try to help people to understand the law and give them a second chance, and then if they do it again we will use the law. Where minor crimes are committed, we never use the law. In Vietnam we consider the relationship between the police and the community the most important so we only use the law if needed. This is the reality in law enforcement. Sometimes minor infringements are not reported.* (Ward Police Chief, District B)

**Figure 5:**
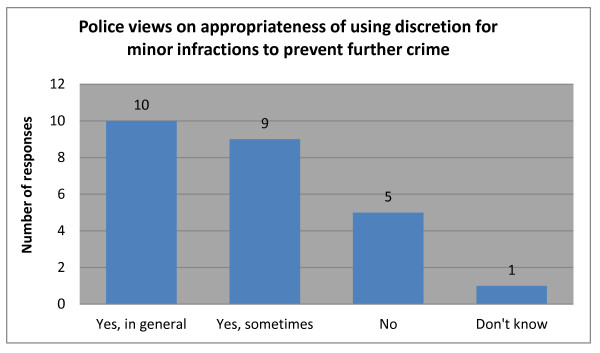
Police views on appropriateness of using discretion for minor infractions to prevent further crime.

The question of discretion, its use and purpose, was also put to police in the survey. Responses in Figure 5 show an overwhelming endorsement of using discretion for less serious matters as a means of discouraging further criminality.

It was interesting that the obligation placed on ward police to get to know each individual in the area had an unexpectedly positive impact on police perceptions of drug users; a degree of empathy developed through this familiarity, as is evident from the following quote.

As a student I thought all drug users were bad people and we had to stay away [from them]. I was scared of them all. But after I joined the police, I had to talk and deal with the drug users … All the time I have to keep an eye on the drug users, and now I feel normal about them, not scared. I’ve concluded that not all drug users are bad people because there are so many different reasons why they take drugs, like family break ups or peer pressure.

### Influences on the engagement of ward police for harm reduction advocacy and training

Interviews with police from the international co-ordination arm of MoPS indicated that ward police were not considered as important actors in the realm of harm reduction. This perception may be influenced by the Ordinance on Commune Police 2008 which refers to the ward/commune police as a ‘part-time armed force in the organizational system of the People’s Police’ (Article 3) [20].

*At the ward level the police are not really police - they are like half police. They deal with social order, they don’t have weapons and they are not professional police … The ward police get information and know about the drug users in their area. The ward police have not had training to be police. They don’t go to the Academy.* (Central level Police Officer A)

Given that limited resources are available for harm reduction advocacy and training among police, priority is usually given to those at senior levels, with the spoken or unspoken assumption that information and policy will trickle down. A police advisor to a major internationally-funded advocacy project targeted at the law enforcement sector in several sites in Vietnam stated that ward police were not included.

*No, [the ward police] are not trained. They might find out about harm reduction by reading the law but I don’t know. I don’t know how they would learn about it. So far we have concentrated on the provincial level and maybe some police at the district level. We would like to train ward police but it is very difficult to train so many police. Maybe in the future we will plan to train ward police, but not yet.* (Central level Police Officer A)

Interviewees reported that not only was there a lack of police training in harm reduction at the ward level, but also that it is unclear how district and higher level police, while aware of harm reduction, actually disseminate that knowledge to enable harm reduction approaches to be implemented by police in the field. As the following quote demonstrates, it appears that ward police are left to rely only on written guidelines, which may be inadequate for concrete decision-making.

*The HIV/AIDS Steering Committee of Hanoi…organises training for the district level [police], but just for the leaders and the commanders, and those in key roles … We have a guidance book in every ward police station. They will have this for MMT and they already have it for NSP; these were sent out to every ward police station.* (District B Police Chief)

The Ordinance on Commune Police 2008 clearly indicates that the specified tasks of the ward police make them appropriate targets for harm reduction information. Perceptions of organisational recruitment and training procedures, however, may impact upon perceptions of the ward level police capability in executing their duties, as the following quote suggests.

*After I met the ward police they were much smarter than I expected even though they only went to the Police College.* (Central level Police Officer B)

### Policy dissemination and communication within the police organisation

We found an inconsistent approach to policy dissemination within the law enforcement sector. Policy directives or official letters are common ways in which senior police communicate policy information to the ward level. According to a Vietnamese UN agency researcher, the provincial level will disseminate a law, policy document or letter but “the documents are normally simplistic, with not much detail,” leaving it up to the subordinate levels to interpret and implement. This respondent went on to say that “a good leader will think of the local context,” and revise the document to include greater detail, but if the leader is lazy or does not support the policy he/she will forward the document down the hierarchy without providing further guidance for localised implementation.

Due to a lack of access to technology at ward police stations, dissemination of information often relied upon fax or face-to-face meetings.

*[District level] Counter-narcotics police are allocated maybe three wards each and they have to keep in touch and share information with their wards. The Counter-narcotics police work at the district level and they go to the wards just for meetings … Every month the Counter-narcotics police have a meeting with the ward police to share information about drugs and to tell them what is going on in the ward.* (Central level Police Officer B)

While some district level police had frequent contact with ward police, those interviewed were not aware of any communication about harm reduction programs or approaches. When asked about how harm reduction training or advocacy targeted at police is communicated to the lower levels of the hierarchy, the director of an internationally-funded harm reduction project in Vietnam responded, “We don’t know. This is a problem. Our project only works at the Central and Provincial level.” A former Central level police officer also indicated that there was uneven communication, at best, regarding harm reduction within the policing organisation.

*When I was training, it would only be one person from the provincial level. I don’t think they would have passed on the information down to the ward level, maybe just among some people in their division.* (Former Central level Police Officer)

According to a Vietnamese NGO researcher, harm reduction advocacy in Vietnam has typically targeted senior police and authorities, without any mechanisms to reach lower levels. The resulting lack of awareness among lower level police meant a continuation of the traditional drug control approaches.

*Maybe at best some police at the district level know [about harm reduction] but I don’t see any effort to go down to the lower level. [The senior levels of police] don’t collaborate and so [the ward police] do their things like drug control and arrest drug users and lock them up to keep the streets clean or they put them in detention or prison.* (Vietnamese NGO researcher)

## Discussion

This study, conducted between 2010 and 2012, reviewed secondary sources and conducted stakeholder interviews and a survey with police to identify the nature and impact of harm reduction advocacy at ward level in urban Vietnam. Given the fact that the survey sample is small and interviewee responses impossible to verify for accuracy, our observations and conclusions are couched in general terms. Despite these limitations, the consistency of responses and the use of multiple methods (including secondary sources) offers some confidence that our findings address gaps in understanding of the critical role played by grassroots police in HIV prevention.

Official HIV/AIDS policy in Vietnam, expressed through the National Strategy on HIV/AIDS [1] and Decree 108 2007, explicitly states that ward police have a key role in HIV prevention; however, to date it appears there has been a lack of systematic inclusion of ward police in harm reduction advocacy strategies to garner their support, or in training to assist them to operationalise these new approaches.

Drug law enforcement and, particularly, crackdowns against drug users, are common practice in Vietnam. These instil fear in drug users about being caught carrying injecting equipment [21]. Legalisation of harm reduction interventions through the 2006 HIV/AIDS Law remains in competition not only with official drug control laws, but operational police cultures and practices which influence police behaviour. The provision of harm reduction services such as NSPs and methadone have been undermined by their use as opportunities for intelligence gathering for the purposes of drug law enforcement, rather than public health. Despite the view that drug control was their primary responsibility, ward police reported exercising considerable discretion for a variety of reasons. These included maintaining good relationships with the community through demonstrating a level of latitude. At present there is little evidence that discretion is explicitly used to support harm reduction approaches; however, its existence as a strategy offers a potential avenue for its use in this regard. Given the international data showing that punitive policing contributes to risk-taking among IDUs – and thus, possible spread of HIV – it is critically important that the official endorsement of harm reduction as an HIV-prevention vehicle be disseminated to all levels of policing.

Our study found that even though key harm reduction programs have a legal foundation in Vietnam, police openly acknowledged engaging in activities such as monitoring pharmacies to identify IDUs accessing clean NS which has been shown to discourage uptake of these programs. Clearly, public policy alone is insufficient without active mechanisms for its dissemination, acceptance and uptake. In the absence of such mechanisms, particularly at street level, other influences act as counterweights to policing. These include the quota system for compulsory detention, internal police disciplinary action and performance review based on the primacy of drug control.

In her study of Australian policing among visible (racial) minorities, Chan argues for the need to “consider our state of knowledge about change” among police ([19], 1), and better understand police organisations and cultures. Although Chan states that her results do not produce a manual for change management due to the inherent complexities of police culture, she highlights the need for greater awareness of the “…contingencies and vagaries of reform” ([19], 65). Central to her argument is the dearth of theories to help understand police culture, which has hindered the ability to answer important questions regarding why some policy reforms “…often make little difference to police practice” ([19], 65). Chan describes a framework which draws on the social theory of Bourdieu and Wacquant [22] and leads her to focus on the role of police as ‘actors’; within this framework, the dynamic nature of police cultures and behaviours are highlighted. She proposes that the outcomes of police practice do not necessarily follow a linear progression but are interactive, where the structural or environmental conditions surrounding police work interact with ‘cultural knowledge’, or the manifestation of personal experience that individual police apply to their police work. The interaction of these factors means that police practice is not necessarily predictable, and that reform or behaviour change can be easily stymied at both the organisation and individual level.

Ward police could be regarded as street-level bureaucrats in the language of Lipsky’s typology of the process of policy implementation [23]. His street-level bureaucrat plays an integral role in determining the extent to which enactment of policy at the grassroots remains consistent with its intent. It is generally accepted that the more layers of bureaucracy the smaller the likelihood of this consistency [24]. The dearth of harm reduction advocacy and training at ward level in Vietnam clearly presents challenges to the survival of both the spirit and letter of new policies in the practices of street police.

As our research findings have demonstrated, ignoring the role of ward police as important actors in policy implementation could further thwart the implementation of harm reduction policies as envisaged by the National Assembly of Vietnam. Ward police, due to their intimacy with local populations, are ideally placed to negotiate the delicate balance between their obligations to deter criminality, ensure safety, and promote effective HIV prevention strategies. Their potential to exercise discretion in this sphere represents a lost opportunity if ward police are not armed with current knowledge about the efficacy and likely public security advantages of harm reduction measures, as well as about their legitimate role in safeguarding and promoting these measures. It is unfortunate and paradoxical that the relatively lower status of ward police in the law enforcement hierarchy has appeared to result in their tacit exclusion from active advocacy and training.

Whilst it is desirable to have harm reduction covered as part of formal police recruit training, it has been argued that ongoing training is more important for the deeper consolidation of particular policing practices [25]. For example, some research has found that recruit academy training can foster police support for policies such as community policing [25] and racial sensitivity [26], but that favourable attitudes dissipated among graduates following exposure to the realities of police work, and police organisation and culture. Haarr [25] argues that formal training must be succeeded by on-street training of approaches learned in the classroom. These observations are likely to have relevance for our area of study.

Project managers, researchers and harm reduction staff must better understand internal police organisational structures and cultures to identify the most effective and efficient strategy to inform and engage the law enforcement sector in Vietnam. The results of this study suggest a range of strategies may be relevant, from capacity building for better internal dissemination systems, to advocating with senior levels on behalf of training for street police on the basis of their potential role in facilitating or inhibiting harm reduction programs. However, it is also essential, based on our findings, that the apparent ambiguities and contradictions between laws to control HIV and to control drugs be discussed more openly in order to clarify the primacy of each for street-level police.

### Consent

Written informed consent was obtained from the patient for publication of this report and any accompanying images.

## Competing interests

The author(s) declare that they have no competing interests.

## Authors’ contributions

MJ was primary researcher conducting field research, analysis and writing this paper; NC and MM responsible for supervision of research, design and writing; and, GM responsible for liaison, co-ordination and writing. All authors read and approved the final manuscript.
